# Empirical evidence of fixed and homeostatic patterns of polyploid advantage in a keystone grass exposed to drought and heat stress

**DOI:** 10.1098/rsos.170934

**Published:** 2017-11-22

**Authors:** Robert C. Godfree, David J. Marshall, Andrew G. Young, Cathy H. Miller, Sarah Mathews

**Affiliations:** Australian National Herbarium, CSIRO National Research Collections Australia, GPO Box 1700, Canberra, Australian Capital Territory 2601, Australia

**Keywords:** polyploidy, fitness, restoration, extreme event, homeostasis, climate adaptation

## Abstract

A long-standing hypothesis in evolutionary biology is that polyploid plants have a fitness advantage over diploids in climatically variable or extreme habitats. Here we provide the first empirical evidence that polyploid advantage in these environments is caused by two distinct processes: homeostatic maintenance of reproductive output under elevated abiotic stress, and fixed differences in seed development. In an outdoor climate manipulation experiment using coastal to inland Australian populations of the perennial grass *Themeda triandra* Forssk., we found that total output of viable seed in drought- and heat-stressed tetraploid plants was over four times higher than in diploids, despite being equal under more favourable growing conditions. Tetraploids also consistently produced heavier seeds with longer hygroscopic awns, traits which increase propagule fitness in extreme environments. These differences add to fitness benefits associated with broader-scale local adaptation of inland *T. triandra* populations to drought stress. Our study provides evidence that nucleotypic effects of genome size and increased reproductive flexibility can jointly underlie polyploid advantage in plants in stressful environments, and argue that ploidy can be an important criterion for selecting plant populations for use in genetic rescue, restoration and revegetation projects, including in habitats affected by climate change.

## 1. Introduction

As a driver of reproductive isolation, phenotypic diversity and speciation, polyploidy has had an unparalleled role in generating plant diversity [1–4]. Polyploids are often widely distributed and their tendency to be common in fluctuating environments [2] has been a matter of interest since the 1930s and 1940s [4–5]. The causes of their success remain under debate [6,7]. Changes in DNA content may alter key ecological traits such as seed size by changing the size, number and geometry of cells [8–10]. Changes in gene copy number may increase biochemical flexibility and promote homeostasis in stressful environments by enabling spatial and temporal partitioning of gene expression among homeologues [3,11] or, in the case of autopolyploids, via maintenance of allelic diversity across loci on homologous chromosomes [12]. Genetic consequences such as the occurrence of transgressive phenotypes [6], chromosomal rearrangements, the masking of deleterious alleles and changes to DNA methylation [13] may also contribute to polyploid advantage in extreme habitats [2]. These can occur rapidly in allopolyploids, and may become important in autopolyploids over time [2].

Understanding how polyploidy enhances plant fitness in stressful environments is of immense practical as well as theoretical significance. As climate change threatens the stability and persistence of vegetation globally, the ability to improve the adaptive capacity of existing or restored plant populations via assisted gene flow [14] or the selection of provenances based on their expected performance under projected climate regimes [15] is seen as a crucial link in the maintenance of biodiversity and ecosystem services. Despite its prevalence in natural plant populations [4–5], however, variation in ploidy has rarely been used as a basis for selecting pre-adapted genotypes for conservation work [16].

**Figure 1.**
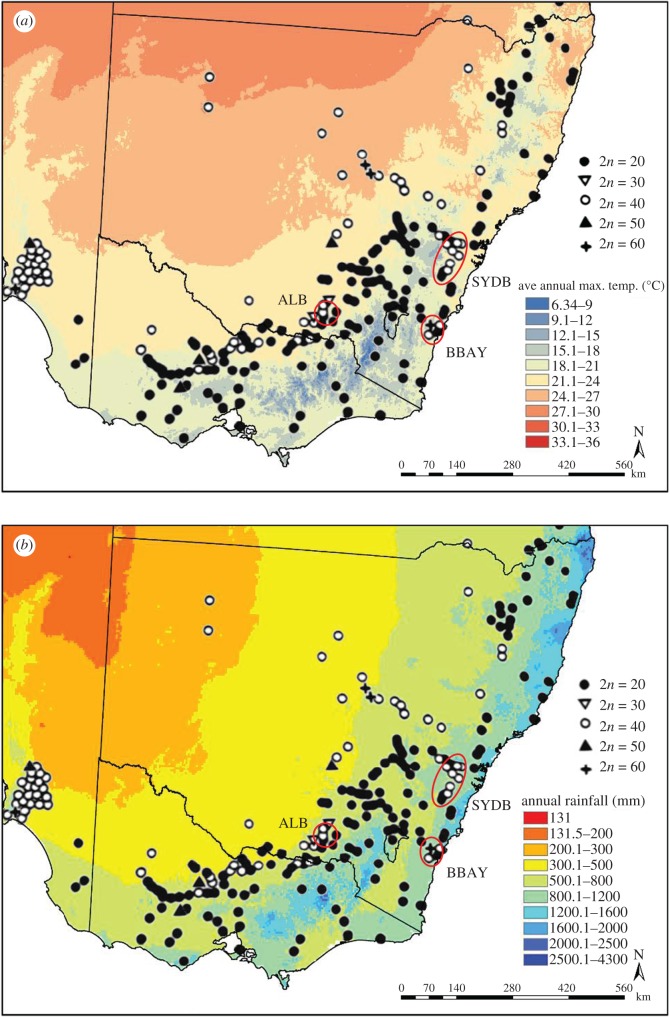
Distribution of *T. triandra* cytotypes in southeastern Australia, with source regions for experimental populations. (*a*) Average annual maximum temperature (°C), showing replacement of diploid cytotypes by tetraploid cytotypes with increasing warmth. (*b*) Total annual precipitation (mm), with tetraploids dominating drier areas. Source regions for population used in the experiment, circled in red, are Bateman's Bay (BBAY), Sydney Basin (SYDB) and Albury (ALB). Cytotypic distribution of *T. triandra* adapted from Hayman [18]. Cytotypes are as follows: diploid 2*n* = 2*x* = 20, triploid 2*n* = 3*x* = 30, tetraploid 2*n* = 4*x* = 40, pentaploid 2*n* = 5*x* = 50 and hexaploid 2*n* = 6*x* = 60.

*Themeda triandra* is a tussock-forming grass native to Africa, Asia, Australia and the Pacific. It is a dominant, ecologically keystone species in Australia, where it is widely used in restoration projects [17]. In Australia, *T. triandra* provides an ideal system for investigation of polyploid advantage, because the species exhibits a striking pattern of continental-scale cytogeographic segregation, with diploids (2*x*; 2*n* = 20) dominating cooler (figure 1*a*) and wetter (figure 1*b*) ecosystems of the southeast, and tetraploids (4*x*; 2*n* = 40) dominating elsewhere. A rapid cytotypic transition occurs along the western slopes of the Great Dividing Range of eastern Australia, coinciding with a shift from wet temperate to seasonally dry or subhumid climates that drives major changes in vegetation, soil and agriculture.

Our objective was to quantify the specific relationships between ploidy, population fitness and climatic stress in *T. triandra* by subjecting diploid and tetraploid populations sourced from across this transition area to experimental drought and atmospheric warming. In particular, we specifically tested whether the tendency for tetraploids to dominate more extreme, hotter and drier environments might be associated with (i) a greater ability to maintain reproductive output under heat and drought stress and (ii) seed characteristics that confer an advantage in extreme environments. We also tested whether any detected fitness advantage was consistently expressed in tetraploids sourced from different parts of the species range.

## 2. Material and methods

### 2.1. Study species

*Themeda triandra* Forssk. is a large, tussock-forming C4 perennial grass that has an extremely broad global distribution, extending from southeast Asia to Australia, India, the Middle East, and southern and eastern Africa. In Australia, *T. triandra* occurs in all mainland states and Tasmania, where it dominates many subtropical and temperate grasslands and savannahs [18]. It is of particular importance in temperate grasslands and grassy woodlands in southern and eastern Australia, many of which are now endangered and of high conservation value [19]. In these systems overgrazing by livestock, application of fertilizer and agricultural intensification tend to drive the replacement of *T. triandra* with introduced annual and perennial herbs and less palatable native species, and intact assemblages are now rare.

In many Australian and African grassland ecosystems, *T. triandra* is considered a keystone species [17], since it plays a disproportionately large role in maintaining herbivore populations and in determining the structure and composition of associated plant assemblages. For example, the ability of *T. triandra* swards to regulate soil nitrate cycling increases the ability of native grasslands to resist invasion by nitrophilic annual weeds, thus maintaining overall ecosystem integrity [20]. Consequently, *Themeda* has become a key element of restoration projects conducted in these ecosystems [21], and it is increasingly being used for other applications, including large-scale rehabilitation of mined sites [22].

In Australia, *T. triandra* exists as a polyploid complex based on *n* = 10. Detailed cytogeographical work by Hayman [18] showed that diploid (2*n* = 2*x* = 20) populations occur in mesic coastal, tableland and slope environments of southeastern Australia, while polyploids are generally found in more xeric inland areas. Among polyploid plants sampled, Hayman [18] found that approximately 95% were tetraploid (2*n* = 4*x* = 40), with a few triploid, pentaploid and hexaploid individuals occurring within diploid or tetraploid populations. On the basis of the high frequency of multivalents in tetraploid cells and the lack of sister taxa in Australia, he argued that the simplest explanation for the origin of tetraploid Australian races of *T. triandra* was through autopolyploidy (despite this being thought to be rare at the time), or through hybridization between different diploid ecotypes, leading to segmental allopolyploidy. Studies of *T. triandra* from South Africa and India suggest that polyploid complexes in these areas are also made of various closely related homologous and homeologous genomes derived from divergent diploid biotypes (viz., segmental allopolyploids [23]). In Australia, the presence of naturally occurring (fertile) triploid and aneuploid swarms and the apparent hybridization of open-pollinated diploid and tetraploid plants suggest that some fertility among diploid and tetraploid races remains [18].

### 2.2. Identification and collection of source *Themeda triandra* populations

Diploid (2*x*) and tetraploid (4*x*) plants used to establish experimental *T. triandra* populations were collected from three different source regions in southeastern New South Wales (NSW) where both cytotypes occur: the Bateman's Bay area (BBAY), the western Sydney Basin (SYDB) and north of Albury in southern NSW (ALB; figure 1*a*). These source regions span three distinct bioregions [24], and experience coastal (BBAY) to seasonally dry warm temperate (ALB) climates (electronic supplementary material, table S1). *Themeda triandra* is common in natural and roadside vegetation in all three collection areas [25–27]. By collecting 2*x* and 4*x* plants from each source region, we avoided confounding source region climate with ploidy level (figure 1) while still allowing for quantification of broader climatic differentiation across source regions. Tetraploid populations from BBAY and SYDB are currently geographically isolated by more than 100 km from those occurring to the north and west (figure 1).

Original collections were made at 38 sites during May to September 2013 (BBAY = 10, SYDB = 18, ALB = 10). At each site, we removed a sample of fresh leaves from 5 to 10 separate plants (at least 1 m apart) which we then transported to the laboratory on ice. We then used flow cytometry, following Doležel *et al*. [28], to determine the ploidy of at least three plants from each site (for full flow cytometry methods, see electronic supplementary material, methods (*a*)). The ratio of peak mean FL2 value of *T. triandra* and the *Pisum sativum* L. ‘Citrad’ standard was close to 0.2 for diploids and 0.4 for tetraploids, with 2C genome sizes of 2*x* and 4*x* plants averaging 1.87 pg (range 1.81–1.93 pg) and 3.66 pg (range 3.57–3.74 pg) respectively (electronic supplementary material, figure S1). Ploidy was confirmed using chromosome counts and by measuring pollen diameter (see electronic supplementary material, methods (*b*)); data for 2*x* and 4*x* cytotypes were consistent with those reported in Hayman [18].

We then randomly selected two 2*x* and two 4*x* populations from each of the three source regions (*n* = 12 populations in total; electronic supplementary material, table S1) for final collection of experimental plants. Returning in October and November 2013, we collected approximately 25 cm^2^ sections from 40 to 50 large mature individual tussocks (no seed was available), removing roots and above-ground foliage. Tussocks were cut back to a common size, transplanted into 20 cm diameter plastic pots containing a low nutrient potting mix suitable for native grasses, watered, and placed into a 25/15°C day/night glasshouse. In February 2014, sections of each tussock of equal size (4 cm basal diameter, 5 cm tall, 10 cm root length) were removed and potted in 20 cm pots. In April 2014, these were moved to outdoor benches where they were watered, fertilized and maintained until July 2014. The ploidy level of all plants was confirmed using flow cytometry, and 24 healthy tussocks from each population (*n* = 24 × 12 = 288 plants total) were randomly selected for use in the field experiment.

### 2.3. Establishment of field experiment

**Figure 2.**
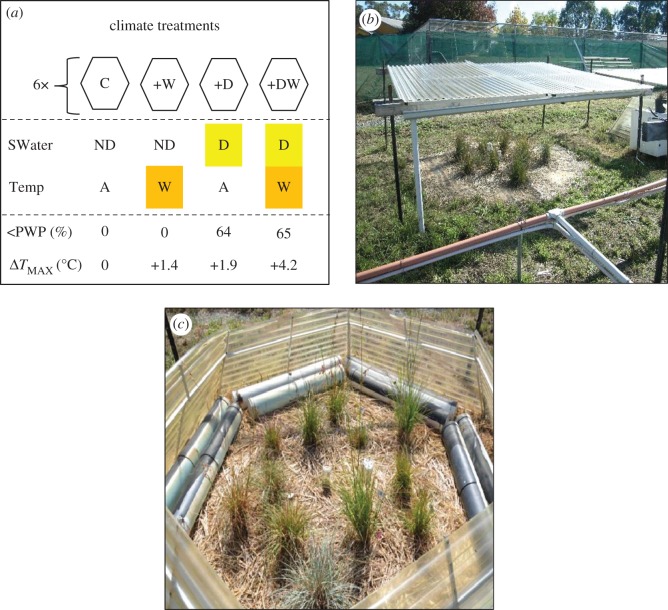
Design of field experiment and climate treatments. (*a*) Plots received both a soil water treatment (SWater), either ND = non-drought or D = drought, and a temperature treatment (Temp), either A = ambient or W = warming, resulting in four climate treatments: C = ambient temperature + non-drought (‘control’), +D = ambient temperature + drought (‘drought’), +W = atmospheric warming + non-drought (‘warm’), and +DW = atmospheric warming + drought (‘warm drought’). Observed soil water and temperature conditions during the experiment are shown: <PWP(%) = the percentage of time with soil water below the permanent wilting point between 1 November 2014 and 10 April 2015 and Δ*T*_MAX_(°C) = change in mean daily maximum temperature in +W, +D and +DW treatments relative to the C treatment. Climate treatments were randomly allocated to plots (*n* = 4) arranged in complete blocks (*n* = 6). (*b*) Polycarbonate rainout shelter used to exclude rainfall and generate drought. (*c*) Polycarbonate hexagon open-top chamber containing water-filled PVC pipes used to generate diurnal and nocturnal atmospheric warming.

The field experiment was conducted in a permanent 800 m^2^ field site located at the CSIRO Black Mountain Laboratories, Canberra, Australia (S 35.27° E 149.11°) between 1 August 2014 and 10 April 2015. The field site consisted of 24 plots arranged into six blocks of four (figure 2*a*). Plots were hexagonal (to be compatible with open-top chambers (OTCs); see below) with an area of 2.2 m^2^ and surrounded to a depth of 60 cm by black plastic, which prevents lateral movement of water into the soil profile [29]. The field site was also serviced by a subterranean drainage system that removes water from the soil profile and from rainout shelters during rainfall events.

Twelve *T. triandra* plants, one from each source population (i.e. six 2*x* and six 4*x* plants), were planted into each plot in late July 2014. Each was removed from their pot, trimmed to common size (4 cm basal diameter), and then placed into a 25 cm diameter and 50 cm deep hole lined with fine flywire (to prevent root competition), backfilled with plot soil, and watered. Plants were arranged in two rows (inner and outer) of six plants each, with all plants having equal growing space. Loose straw, 2 cm deep, was placed around plants to stop soil erosion. Plants were then watered regularly and by October 2014 all experimental plants were healthy and actively growing.

### 2.4. Climate treatments

Between 1 November 2014 and 10 April 2015, whole plots were experimentally subjected to one of four experimental climate treatments: ambient temperature + non-drought (C), ambient temperature + drought (+D), atmospheric warming + non-drought (+W) and atmospheric warming + drought (+DW; figure 2*a*). The C treatment was conducted under non-limiting water availability and ambient temperature conditions typical of the field site in Canberra. During the experiment ambient monthly mean maximum and minimum temperature anomalies at Canberra airport (latitude = −35.3°, longitude = 149.2°) were −0.8 to +4.8°C and −1.4 to +1.5°C, respectively. In the +D treatment plants were subjected to decile 1 or lower precipitation for the treatment period (see below). The +W treatment was intended to simulate projected future warming (*ca* 2050; approximately 2°C) but with non-limiting water availability, while the +DW treatment was intended to simulate drought combined with *ca* 2050 warming.

Each of the four treatments was randomly allocated to one plot within each of the six experimental blocks arranged across the study site in a randomized complete block design. Drought was imposed (+D and +DW treatments) by excluding natural precipitation using clear polycarbonate rainout shelters (figure 2*b*; see [30]). The drought treatment was not confounded with light availability, since rainout shelters were placed on all plots unless the period of rainfall was brief or occurred mainly overnight. Over the treatment period C and +W treatments received a total of 769 and 779 mm of precipitation, respectively, by a combination of rain and hand watering (electronic supplementary material, table S2). In contrast, +D and +DW treatments received 87–88 mm of precipitation (electronic supplementary material, table S2), which was lower than the decile 1 rainfall for the period 1 November –10 April at all collection sites (electronic supplementary material, table S1). Total evapotranspiration slightly exceeded precipitation in all treatments.

Soil water was measured hourly at the near surface (10 cm depth) and at approximately two-week intervals at depths of 10, 20, 30, 40, 50, 60 and 70 cm (full details in electronic supplementary material, methods (*c*)). Prior to the establishment of treatments, no significant differences in volumetric soil water content (SWC_VOL_) were observed between plots assigned to future treatments (*p* > 0.05; electronic supplementary material, figure S2*a*). Following the establishment of the drought treatment on 1 November 2014 SWC_VOL_ at 10 cm depth fell rapidly in +D and +DW plots, and by late November 2014 was approaching the −1.5 MPa permanent wilting point (PWP) (electronic supplementary material, figure S3*b*). Drying of the soil profile at this time occurred to a depth of 30 cm (electronic supplementary material, figure S2*b*). SWC_VOL_ then stayed below the PWP for 64–65% of the entire 161-day treatment period (electronic supplementary material, figure S2*c*), with minor peaks caused by occasional hand watering to prevent drought-stressed plants from dying, and from small rainfall events (electronic supplementary material, figure S3*b*). SWC_VOL_ in non-drought plots was maintained above the PWP, and usually close to the drained upper limit, for the entire treatment period (electronic supplementary material, figure S3*a*).

Passive atmospheric warming was generated in +W and +DW treatments using large polycarbonate hexagon OTCs which generate daytime warming by trapping longwave radiation inside the plot and nocturnal warming via the thermal mass effect of water-filled stormwater pipes positioned inside the OTC (figure 2*c*) [29,30]. Full details on the atmospheric warming, soil warming and atmospheric relative humidity generated in each treatment are provided in electronic supplementary material, table S3 and methods (*d*). Briefly, OTCs (+W treatment) raised average maximum air temperatures by 1.4°C above ambient levels (figure 2*a*), with the greatest warming in November to January. Soil drought (+D) raised average maximum air temperatures by 1.9°C (figure 2*a*), although this difference increased over time (electronic supplementary material, table S3), indicating a rising impact of radiative heating from the drying soil profile [31]. Maximum temperatures in the +DW treatment showed the greatest mean warming (+4.2°C; electronic supplementary material, table S3), and reached an extreme of 51.2°C in January 2015, compared with 44.9°C in the C treatment. Spot measurements (electronic supplementary material, methods (*d*)) showed a similar pattern in midday (11.00–13.00) soil temperatures (5 cm depth), with approximately 2°C warming in +D plots and approximately 1°C warming in OTCs (cf. [30]). Atmospheric relative humidity was higher in C and +W treatments (38.0% and 37.4%, respectively) than in +D and +DW plots (35.6% and 35.9%).

### 2.5. Drought stress

Plants were assessed for signs of drought stress at midday on 18 December 2014, 16 January 2015 and 26 February 2015. We developed a drought stress score (DSS), specific to *T. triandra*, with scores ranging from 0 (no leaf wilting or folding observed, no change in leaf colour) to 5 (all tissue senescent or dead; full details in electronic supplementary material, figure S4). Plants in both +D and +DW treatments were moderately to severely drought stressed on all survey dates (scores of 0.9–1.5 and 1.4–2.0, respectively (electronic supplementary material, table S4)), with loss of turgor, leaf folding along the midrib and leaf dulling. DSS values were consistently around 0.5 units higher in the +DW treatment than the +D treatment. There were no consistent differences in DSS scores between source regions or across cytotypes (electronic supplementary material, table S4).

Supplementary measurements were also taken of abaxial leaf stomatal conductance (SC) using a Decagon SC-1 Leaf Porometer (Decagon Devices, Inc., Pullman, WA, USA), leaf relative water content (LRWC) [32] and pre-dawn leaf water potential (*ψ*_PD_) using a pressure chamber (SoilMoisture Equipment Corp., Santa Barbara, CA, USA). SC, LRWC and *ψ*_PD_ plants in C and +DW treatments were 367 versus 122 mmol m^−2^ s^−1^, 0.84 versus 0.65 and −0.84 versus −1.59 MPa, respectively. Plants in the +DW treatment recorded a DSS of 2.1 when measurements were taken, confirming the stomatal closure, low leaf water content and sub-wilting point leaf water potential measured in drought-affected (+DW) plants.

### 2.6. Collection of growth and reproduction data

Nine variables related to plant reproduction and growth were measured during the treatment phase of the experiment (table 1). On 20 November 2015, when SWC_VOL_ in +D and +DW plots was approaching the PWP and plants were showing signs of water stress, we recorded the basal tussock diameter (mm) of all plants using digital callipers with leaf and stem tissue lightly compressed. At the end of the experiment in April 2015, we harvested all reproductive culms and transported them to the laboratory in paper bags. For each plant, we counted the number of reproductive florets (FLOR_CULM_); i.e. those containing a mature diaspore or dispersal unit (here defined as comprising a ‘seed’ and awn, with the ‘seed’ comprising a caryopsis with attached lemma) on up to five culms carefully selected to be representative of the total, and then took a random sample of up to 50 mature diaspores. We then determined the viability of each seed by performing a squeeze test with tweezers (and dissection where needed) to confirm the presence and viability of the endosperm (e.g. [33]); the validity of this approach was confirmed by testing a subset of 100 seeds for viability using tetrazolium staining. For 10 randomly selected viable diaspores, we then measured the length of the awn, stretched straight, and total seed weight with awns removed. We also again recorded the tussock basal area at the time of the final harvest.

**Table 1.** Reproduction and growth of *T. triandra* under experimental climate treatments. Compared with diploids, seed production and seed viability in tetraploids show a homeostatic response to increasing climate stress, with associated models containing strong cytotype × climate interactions (CY × CL). In contrast, the increase in seed mass and awn length observed in tetraploids was fixed across climate treatments, with only cytotypic effects (CY) being significant. Cytotypic differences were consistent across source regions (SY), with CY × SY interactions being non-significant in eight of nine models.

	model effects^a^			
variable	main	interaction	model^b^	figure^c^
viable seed production (dm^−2^)	CY_2.9_^NS^; CL_9.5_***; SR_21.4_**	CY × CL_5.8_***; SR × CL_2.0_^M^	HA	3*a*
viable seed weight (g 10 seeds^−1^)	CY_33.6_**; CL_8.2_**; SR_19.8_**	CY × SR_8.5_*	FA	3*b*
basal area ratio (Apr/Nov)	CY_0.6_^NS^; CL_14.0_***; SR_0.1_^NS^	SR × CL_3.1_**	NA	3*c*
viable seed production (plant^−1^)	CY_5.4_^M^; CL_17.1_***; SR_22.5_**	CY × CL_4.6_**; SR × CL_2.1_^M^	HA	S5*a*
culm production (dm^−2^)	CY_1.4_^NS^; CL_2.3_^NS^; SR_23.2_**	CY × CL_4.3_**; SR × CL_2.5_*	HA	S5*b*
floret production (culm^−1^)	CY_4.6_^M^; CL_12.2_***; SR_17.5_**	CY × CL_2.8_*	HA	S5*c*
seed viability (%)	CY_20.9_**; CL_4.8_*; SR_0.4_^NS^	CY × CL_3.0_*	HA	S5*d*
basal area (Apr)	CY_3.9_^M^; CL_25.8_***; SR_0.9_^NS^	SR × CL_3.6_**	NA	S5*f*
awn length (mm)	CY_29.3_**; CL_9.2_**; SR_36.0_***	CY × CL × SR_3.3_**	FA	S5*e*

^a^CY = cytotype effect (2*x* versus 4*x*), CL = climate treatment effect (C, +W, +D, +DW), and SR = source region effect (BBAY, SYDB, ALB). Only highest order model interaction terms significant at the 0.10 level are shown. Subscripts for main and interaction effects indicate *F* values for fixed effects, evaluated with numerator and denominator degrees of freedom as follows: CY = 1,6; CL = 3,15; SR = 2,6; CY × CL = 3, 178–236; SR × CL = 6, 178–236; CY × SR = 2, 6; CY × CL × SR = 2, 178–236. ***p < 0.001; **p < 0.01; *p < 0.05; ^M^*P* < 0.10; ^NS^*P* > 0.10.

^b^The relevant polyploid advantage model is taken from figure 4: HA = homeostatic advantage, FA = fixed advantage and NA = no advantage.

^c^Refers to either figure 3*a–c* or electronic supplementary material, figure S5*a–f*.

### 2.7. Statistical analyses

We determined the total viable seed production (VSP_TOT_) according to VSP_TOT_ = CULM_PLANT_ × FLOR_CULM_ × SEED_VIAB_, where CULM_PLANT_ is the number of reproductive culms, FLOR_CULM_ is the number of reproductive florets per culm and SEED_VIAB_ is the proportion of viable seed. The final variables used in subsequent statistical analyses were: (i) VSP_TOT_ per whole plant (plant^−1^), (ii) VSP_TOT_ per unit decimeter^2^ (dm^−2^) of tussock basal diameter, (iii) culm production (dm^−2^), (iv) floret count (culm^−1^), (v) seed viability (%), (vi) viable ten-seed mass (g), (vii) awn length (mm), (viii) basal area ratio, based on the ratio of tussock basal areas recorded on 10 April 2015 and 20 November 2014, and (ix) tussock basal area (cm^2^) in April 2015.

Data were analysed using generalized linear mixed model (GLMM) analysis using SAS Version 9 (Carey, NC, USA), with each of the nine variables used as dependent variables. Fixed predictors included cytotype (CY; 2*x* versus 4*x*), climate treatment (CL; C, +W, +D, +DW), source region (SR; ALB, SYDB, BBAY), all two-way interaction terms (CY × CL, CY × SR, CL × SR), the three-way interaction term (CY × CL × SR) and plot location (inner set of six plants versus outer set of six plants). Random model effects included block (*n* = 6), plot within climate treatment (24 plots total across 4 climate treatments), and collection site within cytotype and source region. In two models, the block variable was excluded to ensure model convergence.

Models were constructed using a Gaussian or lognormal response distribution and identity link with Kenward--Roger- or containment-method denominator degrees of freedom. Data were transformed and two outliers where plants or seed had been affected by herbivory were removed to improve compliance with model assumptions. Mean cytotypic and source region differences at each level of climate treatment were evaluated using Tukey–Kramer least square means (LSM) tests. Back-transformed means were determined for diploid (*μ*2*x*) and tetraploid (*μ*4*x*) cytotypes from LSM (note that for ln- and square-root transformed data back-transformed means are not the same as arithmetic means of the original data, despite being on the same original scale), and a measure of polyploidy advantage, *W*_TD_, was determined as the ratio of back-transformed means in the original scale (*W*_TD_ = *μ*4*x*/*μ*2*x*).

## 3. Results and discussion

**Figure 3.**
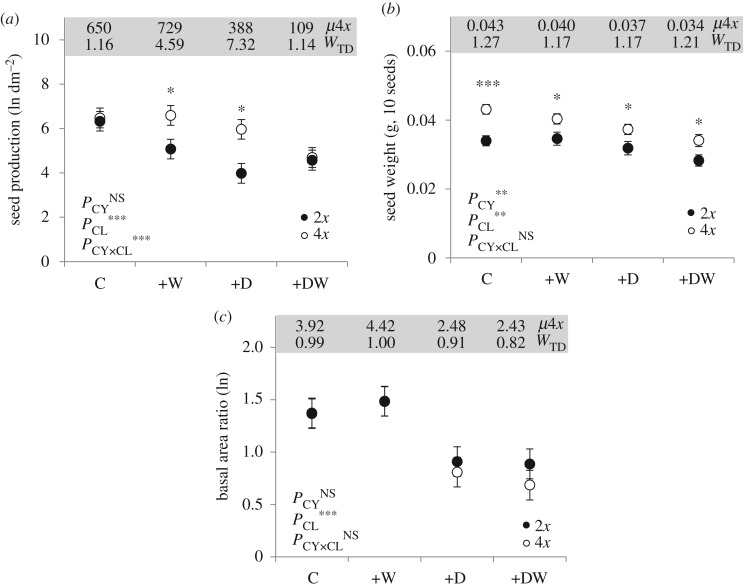
Reproduction and growth in experimental populations of tetraploid (4*x*) and diploid (2*x*) *T. triandra* under experimental drought and atmospheric warming. (*a*) Homeostatic polyploid advantage in seed production (per unit basal area of tussock) in drought and atmospheric warming treatments. (*b*) Fixed increase in weight of viable seeds produced by tetraploid plants across climate treatments. (*c*) Ratio of tussock basal areas after (April 2015) and prior to (November 2014) climate treatment, showing no polyploid advantage. Climate treatments (*y* axes) are as follows: C = control, +W = warm, +D = drought and +DW = warm drought. In (*a*) and (*c*) tetraploid means (*μ*4*x*) are back-transformed from GLMM-derived least-square means (see Material and methods). A measure of polyploid advantage, *W*_TD_, is defined as *W*_TD_ = *μ*4*x*/*μ*2*x* where *μ*2*x* is the mean for diploid plants, again back-transformed in (*a*) and (*c*). *p*_CY_, *p*_CL_ and *p*_CY×CL_ are *p* values of cytotype, climate treatment and cytotype × climate treatment interaction, respectively. Significant cytotypic group mean differences (4*x* versus 2*x*) are shown for each climate treatment. ^NS^*p* > 0.10, *p < 0.05, **p < 0.01, ***p < 0.001.

Tetraploids from all source regions were more fit than diploids, either across all climate treatments (including the control), or only in heat or drought stress treatments. Significant (*p* < 0.05) cytotypic (CY) or cytotype × climate treatment (CY × CL) effects were present in the data for seven fitness traits (table 1). Strikingly, total output of viable seed under heat (+W) and drought (+D) treatments was four to seven times higher per unit basal area in tetraploid plants (figure 3*a*), a response characterized by a strong CY × CL interaction (*P*_CY×CL_ < 0.001; table 1). Higher production of viable seed in tetraploids under heat and drought stress was also evident at the whole plant scale (electronic supplementary material, figure S5*a*). Tetraploids maintained higher culm production, floret per culm production and seed viability in +W and/or +D climates (electronic supplementary material, figure S5*b–d*), thus displaying polyploid advantage in multiple components that impact seed production. Tetraploids also produced significantly heavier seeds (figure 3*b*) with longer awns (electronic supplementary material, figure S5*e*) than diploids, but here the increase in propagule size remained similar across most treatments, with associated CY × CL interactions being weak or absent, and direct cytotypic effects (CY) being strong (table 1). In contrast, tussock growth (figure 3*c*) and basal area (electronic supplementary material, figure S5*f*) did not show significant cytotypic differences.

**Figure 4.**
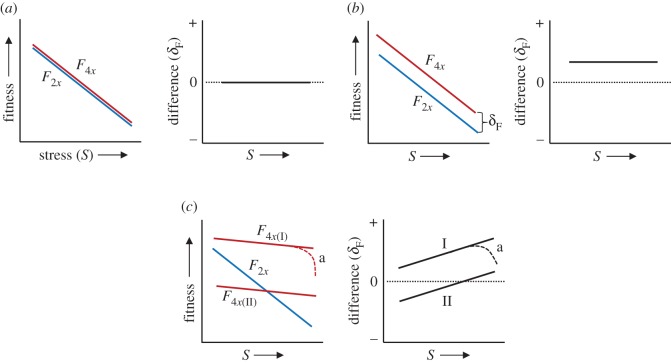
Conceptual models of polyploid advantage under drought or heat stress. Consider a situation in which the fitness of tetraploid (*F*_4*x*_; red) and diploid (*F*_2*x*_; blue) populations declines with increasing drought or heat stress (*S*). (*a*) In the null or *no advantage* (NA) model the fitness of tetraploid and diploid cytotypes is equal (*F*_4*x*_ = *F*_2*x*_) for all *S*. In the NA model, *δ*_F_ = *F*_4*x*_ − *F*_2*x*_ = 0. (*b*) In the *fixed advantage* model, the fitness advantage of tetraploid plants (*δ*_F_) is constant across all levels of *S*. In this model *δ*_F_ = *k,* where *k* is a constant such that *k* > 0 for all *S*. Fixed differences in fitness reflect genetic or ontogenic differentiation across populations that is unaffected by stress. (*c*) Two *homeostatic advantage* (HA) models in which the relative fitness of tetraploid cytotypes becomes higher under increasing stress, such that *δ*_F_ = *kS.* HA can arise from high relative polyploid fitness in all environments such that *F*_4*x*_ > *F*_2*x*_ and *δ*_F_ > 0 for all *S* (*F*_4*x*(I);_ case I) or only in more stressful environments (*F*_4*x*(II);_ case II), the latter driving niche differentiation among cytotypes. Dashed lines ‘*a*’ in HA models show nonlinear loss of polyploid advantage under extreme stress.

These data provide empirical evidence of both homeostatic and fixed advantage (figure 4) in tetraploid plants, suggesting that distinct processes combine to promote polyploid advantage in *T. triandra*. Here we follow Caswell [34] in defining homeostasis as the ability to adjust internal conditions in the face of a changing external environment. A pattern of homeostatic reproductive advantage in tetraploids (cf. figures 3*a* and 4*c*) was indicated by their ability to maximize production of viable seeds under abiotic conditions that severely curtailed the basal growth (figure 3*c*) and size of individual tussocks (electronic supplementary material, figure S5*f*). This suggests they are better able to alter internal allocation of photosynthate during extreme climatic events. Flexible resource allocation is typical of plants adapted to variable or extreme habitats [35], and in polyploids it may be linked to increased metabolic and physiological flexibility afforded by gene subfunctionalization. Indeed, transcriptional and post-transcriptional regulation of hom(e)ologous gene copies [36] and differential gene expression across tissues and organs in response to abiotic stress [11] are thought to increase the ability of polyploids to respond to environmental variability. Whether this and/or ploidy-linked variation in traits associated with water balance and optimal carbon fixation [9,37] are responsible needs to be resolved, but irrespective of the mechanism, the ability of only tetraploid plants to maintain substantial seed production (figure 3*a*) under growth-constraining drought (+D) conditions provides a clear demographic basis for the cytogeographic segregation of tetraploid and diploid cytotypes observed in *T. triandra* at the continental scale (figure 1).

A pattern of fixed reproductive advantage was evident in seed morphological traits. Tetraploids consistently produced approximately 20% heavier seeds (figure 3*b*) with longer hygroscopic awns (electronic supplementary material, figure S5*e*) across climate treatments (cf. figures 3*b* and 4*b*). In polyploids, the tendency for cell size and volume to increase with nuclear DNA content, including the direct gene multiplication *per se* that occurs in autopolyploids [2], frequently manifests as increases in the size of pollen, organs, and seed [8], which in turn have the potential to alter significantly the demography and evolutionary dynamics of plant populations [38]. While the specific relationship between propagule size and fitness in *T. triandra* requires further study, seed size plays a pivotal role in determining seed viability, dispersal, and seedling survival, and large seeds may be particularly advantageous in drought-affected habitats where the ability to rapidly establish deep roots allows seedlings to avoid surface soil drought [39]. Awn length is a heritable trait in some grasses [40] and the longer awns in tetraploids (15% longer on average than those produced by diploids; electronic supplementary material, figure S5*e*) might be advantageous in arid habitats during periods of soil surface drying, when microsites become essential for reproductive success, and following fires, which destroy litter on the soil surface. The geniculate awns are hygroscopic, twisting in response to changes in atmospheric humidity, a morphological adaptation that promotes secondary dispersal of the diaspore over the soil surface to favourable microsites, and that may facilitate subsequent seed burial [41].

Drought-related polyploid advantage in *T. triandra* appears to add to fitness benefits associated with broader local adaptation of populations to rainfall and climate. For example, populations from the hottest and driest source region (Albury; figure 1*a–b*) had a tendency to maintain seed production despite increasing climatic stress (electronic supplementary material, figure S6*a*), and to grow more rapidly in response to high versus low water availability (C and +W treatments in electronic supplementary material, figure S6*b*). Both are responses that facilitate reproductive success in periodically stressful environments. Plants from Albury also typically produced diaspores with the heaviest seed (electronic supplementary material, figure S6*c*) and longest awns (electronic supplementary material, figure S7*a*), both adaptations to dry environments. In contrast, local adaptation appears to have favoured reduced seed production in plants sourced from dry, inland habitats (electronic supplementary material, figure S6*a*). While a trade-off between seed size and number is not unexpected [42], the magnitude of the difference may reflect a tendency for experimental plants from these areas to be generally larger than ones from coastal areas (electronic supplementary material, figure S7*b*), a difference also noted in wild populations.

Collectively, the development of cytotypic and ecotypic differences in reproduction and growth, combined with the strong conservation of cytotypic differences across source regions (i.e. weak or absent source region × cytotype interactions; table 1), indicate that the presence of polyploid cytotypes increases the resilience of regional metapopulations to stressful climatic conditions, and expands the climatic niche of species as a whole. As discussed above, a parsimonious explanation for this pattern is that tetraploids in each source region are derived from non-homogeneous local diploid ecotypes, thus maintaining regional differentiation while gaining a range of traits associated with both increased cellular DNA content and genomic differentiation. However, whether tetraploids from each region represent independent polyploidization events, as argued by Hayman [18], or a single event followed by range extension and subsequent isolation remains to be determined.

Under the most extreme conditions of combined heat and drought (i.e. +DW treatments), *T. triandra* tetraploids showed no advantage in seed production (figure 3*a*). Since soil water availability in +D and +DW treatments was equivalent, the dramatic decline in fecundity of tetraploids in the +DW treatment can be attributed to the 2–3°C increase in daily maximum air temperatures, which resulted in higher drought stress (see Material and methods) especially during the hottest days when extremes exceeded 50°C. The effects of soil drought on plant physiology and mortality are exacerbated by high temperatures [43], and threshold-type mortality responses (figure 4*c*) in drought-affected grassland plant populations are common [44]. Our data indicate that under realistic scenarios of future climate change, the reproductive output of tetraploid populations during drought may lose their advantage and become increasingly susceptible to nonlinear demographic behaviour and rapid genetic and phytosociological change [45].

## 4. Conclusion

The mechanisms by which polyploidy increases the reproductive flexibility and fitness of plant populations in extreme or variable environments have been a long-standing question in ecology and evolutionary biology. Our study provides the first empirical evidence that polyploid advantage in plants in such habitats involves two independent processes: (i) homeostatic maintenance of reproductive output under increasing abiotic stress and (ii) fixed differences in seed size and morphology that increase propagule fitness and mobility. Investigation of the mechanisms through which genome duplication and subsequent evolution have each contributed to the fitness of Australian populations of *T. triandra* is a promising area for future research.

Evidence of polyploid advantage has strong implications for conservation projects that use predictive provenancing and assisted gene flow to future-proof natural or restored vegetation. Very little plant selection for use in conservation work has focused on ploidy level [16], in striking contrast to its pivotal role in agricultural improvement. Our study shows that selection for ploidy could enhance the success of revegetation projects involving *T. triandra* across temperate Australia, and might particularly enhance population resilience during periods of extreme heat and drought [44]. Given the prevalence of ploidy variation within plant species worldwide [2,46], and the growing impact of extreme climate events on plant populations [43], polyploid cytotypes could be a valuable source of adaptive genetic variation for similar projects on a global scale.

## Supplementary Material

Supplementary Figures 1-7, Tables 1-4 and Methods

## Supplementary Material

Supplementary Data

## Data accessibility

The dataset supporting this article have been uploaded as part of the electronic supplementary material.
