# Supplementary material for: Empirical evidence of fixed and homeostatic patterns of polyploid advantage in a keystone grass exposed to drought and heat stress
Source: R Soc Open Sci. 2017 Nov 22;4(11):170934. doi: 10.1098/rsos.170934 (PMC5717662; doi:10.1098/rsos.170934)
Supplement: Supplementary Figures 1-7, Tables 1-4 and Methods [file rsos170934supp1.docx]

**SUPPLEMENTARY INFORMATION**

**Figure S1.** Indicative flow cytometry results and 2C genome sizes of diploid and tetraploid *Themeda triandra*.

**Figure S2.** Soil water profiles based in neutron access probe data.

**Figure S3.** Near-surface (10 cm) volumetric soil water content during the treatment phase of the experiment.

**Figure S4.** Drought stress scores (DSS) developed for *Themeda triandra*.

**Figure S5.** Reproduction and growth in experimental populations of tetraploid (4x) and diploid (2x) *Themeda triandra* under experimental drought and atmospheric warming.

**Figure S6.** Reproduction and growth in experimental populations of *Themeda triandra* from different source regions under experimental drought and warming treatments.

**Figure S7**. Awn length and tussock size in experimental populations *Themeda triandra* under experimental drought and warming.

**Table S1.** Climate data for each of the 12 collection sites used to source *Themeda triandra* populations used in the experiment.

**Table S2**. Water balance data for each climate treatment during the experiment.

**Table S3**. Air temperature data (10 cm) for each climate treatment.

**Table S4**. Mean drought stress scores for plants in each climate treatment.

**Supplementary Methods**

1. Flow cytometry
2. Pollen size
3. Soil water measurements
4. Temperature and humidity

**Figure S1**


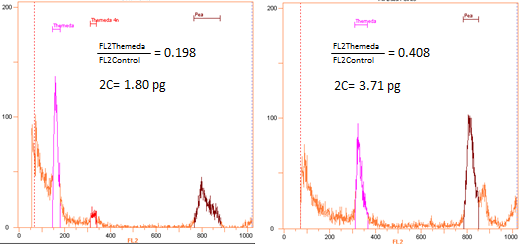


*(a)*

*(b)*

**Figure S1.** Indicative flow cytometry results and 2C genome sizes of diploid and tetraploid *Themeda triandra*. *(a)* diploid; FL2 mean = 161.9, CV = 4.8%. *(b)* tetraploid; FL2 mean = 333.5, CV = 4.1%.

**Figure S2**

**Figure S2.** Soil water profiles based in neutron access probe data. *(a)* Before the application of climate treatments on October 21 2014. *(b)* At the end of the dry-down period on November 21 2014, showing soil drought to 30 cm in +D and +DW treatments. *(c)* During the main treatment phase on December 18 2014, with drought in +D and DW treatments well established to at least 50 cm. Significance values indicate treatment differences at each soil depth; **P* < 0.05, ***P* < 0.01, ****P* < 0.001.

**Figure S3**

**Figure S3.** Near-surface (10 cm) volumetric soil water content during the treatment phase of the experiment. *(a)* C and +W climate treatments. *(b)* +D and +DW treatments. DUL = drained upper limit, approximately 30% vol; PWP = permanent wilting point, approximately 8% vol.

**Figure S4**

**Figure S4.** Drought stress scores (DSS) developed for *Themeda triandra*. *(a)* 0 = no loss of turgor, no leaf folding, no change in leaf colour. *(b)* 1 = loss of leaf turgor with wilting, minor leaf dulling. *(c)* 2 = leaf folding along midrib, major dulling of leaves. *(d)* 3 = complete leaf folding, senescence developing on leaf tips, <50% of leaves. *(e)* 4 = >50% of leaf tissue senescent or heavily bleached. *(f)* 5 = all tissue senescent or bleached; plant approaching death.

**Figure S5**

**
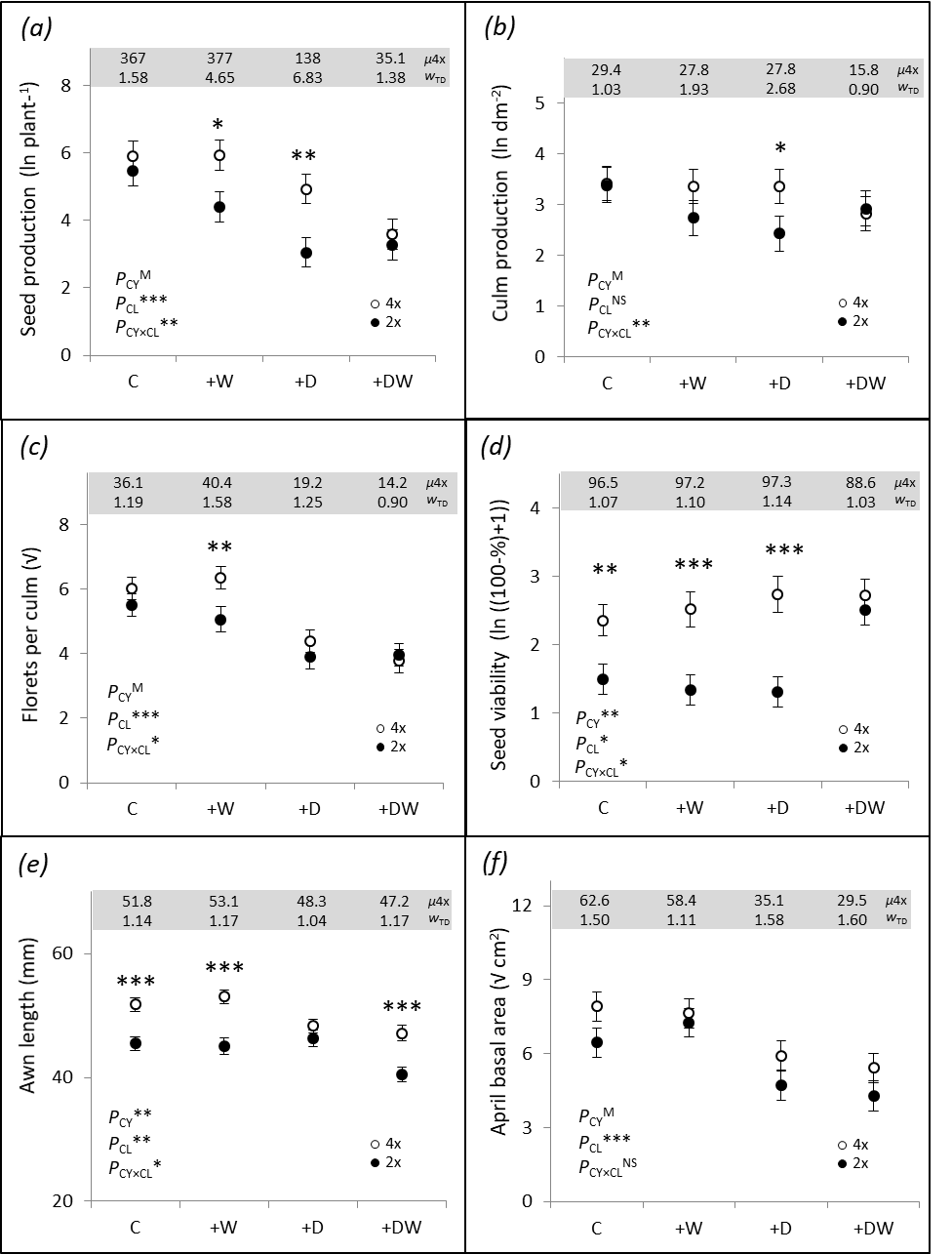
**

**Figure S5.** Reproduction and growth in experimental populations of tetraploid (4x) and diploid (2x) *Themeda triandra* under experimental drought and atmospheric warming. *(a)* Seed production per plant, showing homeostatic seed production in warm and drought treatments. *(b)* Culm production per dm^2^ of tussock basal area. *(c)* Floret production per culm. *(d)* Seed viability; note the reflected transformation of the raw data. *(e)* Awn length of the diaspore. *(f)* Tussock basal area in April 2015, at the end of the experiment. Climate treatments (*y* axes) are as follows: C = control, +W = warm, +D = drought and +DW = warm drought. Tetraploid means (*μ*4x) are back-transformed in *(a)-(d)* and (*f*) from GLMM-derived least-square means. A measure of polyploid advantage, W_TD_, is defined as W_TD_ = *μ*4x/ *μ*2x where *μ*4x is the mean for diploid plants, again back-transformed in *(a)*-*(d)* and *(f)*. *P*_CY_, *P*_CL_ and *P*_CY×CL_ are *P* values of cytotype, climate treatment, and cytotype x climate treatment interaction respectively. Significant cytotypic group mean differences (4x vs. 2x) are shown for each climate treatment. ^NS^*P* > 0.10, ^M^*P* < 0.10, **P* < 0.05, ***P* < 0.01, ****P* < 0.001.

**Figure S6**

**Figure S6.** Reproduction and growth in experimental populations of *Themeda triandra* from Bateman’s Bay (BBAY), Sydney Basin (SYDB) and Albury (ALB) source regions (see figure 1) under experimental drought and warming treatments (*x* axes). *(a)* Production of viable seeds per unit tussock basal area, showing a marginal tendency for homeostasis in plants from the Albury region. *(b)* Ratio of tussock basal areas after (Apr 2015) and prior to (Nov 2014) climate treatment, with increased responsiveness of Albury plants to water availability. *(c)* Weight of viable seeds. *P*_SR_, *P*_CL_ and *P*_SR×CL_ are *P* values of source region, climate treatment and source region x climate treatment interaction respectively. Significant regional mean differences (BBAY vs. SYDB vs. ALB) are shown for each climate treatment. ^NS^*P* > 0.10, ^M^*P* < 0.10, **P* < 0.05, ***P* < 0.01, ****P* < 0.001.

**Figure S7**

**
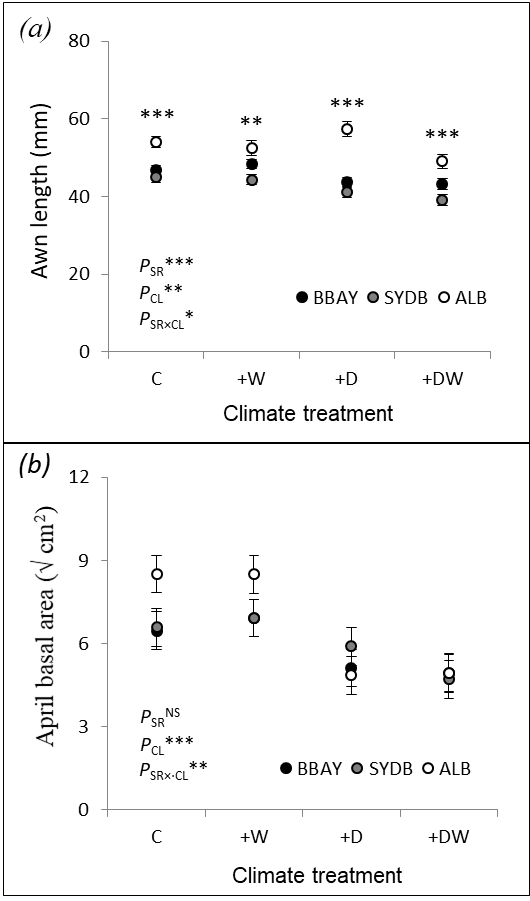
**

**Figure S7**. *(a)* Awn length and *(b)* tussock size in experimental populations of *Themeda triandra* from Bateman’s Bay (BBAY), Sydney Basin (SYDB) and Albury (ALB) source regions under experimental drought and warming. Legend as in figure S6.

**Table S1.** Climate data for each of the 12 collection sites used to source *Themeda triandra* populations used in the experiment. Data in bold (grey cells) are means for each source region. Based on data 1950-2014 sourced from the SILO Patched Point Dataset (www.longpaddock.qld.gov.au).

^1^Tmax_A_ = mean annual daily maximum (°C), ^2^Tmax_Jan_ = mean January daily maximum (°C), ^3^Tmin_A_ = mean annual daily minimum (°C), ^4^Tmin_Jul_ = mean July daily minimum (°C), ^5^TP_A_ = mean annual precipitation (mm), ^6^TET_A_ = mean annual potential evapotranspiration (mm), ^7^TP_N-A_ = total precipitation Nov 1 – Apr 10 (mm), ^8^TET_N-A_ = total potential evapotranspiration Nov 1 –Apr 10, ^9^TP_N-A_(D1) = decile 1 total precipitation for the period Nov 1-Apr 10.

**Table S2**. Water balance data for each climate treatment during the experiment. Profile change indicates the change in total soil water across the soil profile to a depth of 60 cm between November 2014 and April 2015. Precipitation refers to water received either as rain or by watering from hose. ET = evapotranspiration.

**Table S3**. Air temperature data (10 cm) for each climate treatment. Data for C (control) treatment are observed temperatures in °C; data for +W, +D and +DW treatments are change in temperature (ΔT) relative to C. TMAXav = mean daily maximum , TMAXext = extreme monthly maximum, TMINav = mean daily minimum, TMINext = extreme monthly minimum.

**Table S4**. Mean drought stress scores for plants in each climate treatment. Standard errors are in parentheses. DI = diploid (2x), TE = tetraploid (4x).

**Supplementary Methods**

1. **Flow cytometry***.*

Flow cytometry was performed using freshly harvested leaf tissue, either collected from the field and stored on ice, or directly from plants grown in the glasshouse or in the experiment. The method in general follows Doležel (2007)[1]. Approximately 50 mg of leaf tissue was placed in 1ml ice cold Modified Galbraith buffer [2] (4.58g MgCl_2_, 2.1g MOPSO, 4.44g Citric Acid, 15.0g PVP-10, 0.5 ml Triton X-100, 2.5 ml Tween 20. The pH was adjusted to 7.0 – 7.1 with 1 M NaOH and filtered through a 0.22-μm filter and stored at −20°C) in a Petri dish and gently chopped with a razor blade for 30 - 60 seconds to release intact nuclei. It was co-chopped with 40 – 50 mg of an internal standard *Pisum sativum* L. ‘Citrad’ (2C DNA content 9.09 pg DNA). The homogenate was pipetted up and filtered through a 42-um mesh. This was followed by an extra filtering step using a 2 x 22um filters to minimize blockages to flow cytometer. 50ul of Rnase at 1mg/ml and 50ul Propidium Iodide at 1mg/ml was added to the sample, gently mixed and left on ice for a short period (usually less than 10 minutes) before being processed. The samples were analyzed with a Beckman Coulter Cell lab Quanta^TM^ SC flow cytometer equipped with a 488nm laser at 22mW. Histogram data was collected using the FL2 detector and analysis was performed with Beckman Coulter Cell Lab Quanta SC MPL analysis software.

Broad screening of plants for ploidy level was achieved by sampling field- and glasshouse-grown plants from across all source regions (*n* = 567 samples in total) and conducting flow cytometry as described above. For each plant, 250ul of sample was run at 10 to 40uL/sec until measurable peaks were obtained. The peak mean FL2 value of *Themeda triandra* was divided by mean peak FL2 value of standard and the resulting ratio was used to determine ploidy level; ratios were close to 0.2 and 0.4 for diploids and tetraploids respectively (see below). Occasional hexaploids and triploids were observed but only diploid and tetraploid plants were used in the experimental field trial. Additionally, 2C DNA content was carefully determined for one plant from each source population (n = 12). Each plant was sampled a minimum 3 times on 3 separate days for each as described above. 250ul of sample was run at 7 -28uL/s until a total count of 5,000 – 15,000 and a CV of <5% was reached. Histogram data was collected using the FL2 detector with a gain of 3.5 – 4.5 and analysis was performed with Beckman Coulter Cell Lab Quanta SC MPL analysis software.

Mean FL2 values for diploid *Themeda*, tetraploid *Themeda* and *Pisum* were 172.2 (SE = 2.6; range = 165.4 – 186.3; mean CV = 4.7%), 330.5 (SE = 1.5; range = 165.4 – 186.3; mean CV = 3.9%) and 824.0 (SE = 5.9, range = 732.8 - 914.7; mean CV = 2.9%) respectively. Mean *Themeda*/*Pisum* FL2 ratios were 0.206 (SE = 0.002; range 0.199-0.212) and 0.402 (SE = 0.002; range 0.393-0.419) for diploids and tetraploids respectively.

1. Doležel, J., Greilhuber, J. & Suda, J. Estimation of nuclear DNA content in plants using flow cytometry. *J. Nature Protocols* **2**, 2233-2244 (2007).
2. Galbraith, D. W. *et al*. Rapid flow cytometric analysis of the cell cycle in intact plant tissues. *Science* 220: 1049–1051 (1983).
3. **Pollen size**

Flowers were collected from all plants within two control (C) plots, each containing one plant from all 12 source locations. Anthers were dissected out of three different florets, placed in a drop of FLP Orcein stain and macerated using a brass rod. A coverslip was gently placed on top of the sample but not squashed and topped up with more stain if needed. The slides were examined on an Olympus BH-2 microscope under x40 magnification. Random areas of the slide were chosen and any mature pollen grains in the field of view were measured using a graticule scaled at 0.1mm = 40 lines. For each slide the diameter of 10 – 20 pollen grains were measured. The ploidy level of experimental source populations were corroborated using chromosome counts and by measuring pollen diameter on two representative plants from each population: mean diameters were 39.3 μm (range 35.4-44.6 μm) for diploid pollen and 45.5 μm (range 42.8-50.6) for tetraploid pollen. These were consistent with pollen sizes reported by Hayman (1960) [1].

1. Hayman, D. L. The distribution and cytology of the chromosome races of *Themeda australis* in southern Australia*. Aust. J. Bot.* **8**, 58-68 (1960).
2. **Soil water measurements**

Hourly changes in near-surface (10 cm depth) soil water were measured near the centre of four plots (one in each treatment) using a HOBO® S-SMC-M005 soil moisture sensor connected to a H21-002 micro station. Additional measurements of soil at soil depths of 10, 20, 30, 40, 50, 60 and 70 cm were taken at approximately two-week intervals using neutron access tubes and a CPN® 503DR Hydroprobe (CPN International, Inc., Concord, CA, USA). Volumetric SWC was calculated using the calibration equation: SWC_VOL_ = 2.0 × 10^-7^*x*^2^ -1.5 × 10^-4^*x* + 1.82 where *x* = the neutron probe reading, which was determined by comparing the SWC_VOL_ of soil core sections with probe readings taken at the time of removal.

1. **Temperature and humidity**

Air temperatures were recorded hourly in the center of each plot at 10 cm above the soil surface using Hobo® Pendant UA-002-64 data loggers shielded underneath 10 cm pots covered with reflective silver tape. Spot soil temperature data were collected using an YCT YC-821 thermometer with HP-502A-M12 (k) penetration temperature probe between 11 am and 1pm on 18 Dec 2014 and 25 Feb 2014. Soil temperature was recorded at 5 cm depth in four locations (north, east, south and west, half way between plot centre and plot edge). Finally, we recorded atmospheric relative humidity (RH, %) by spot readings taken in the centre each plot between 11 am and 1 pm on 25 February 2015 using a KIMO® AQ200 air quality sensor.

**Additional description of air and soil temperate data**

Between 1 Nov 2014 and 10 Apr 2015 mean daily maximum (TMAX_AV_) and minimum (TMIN_AV_) 10 cm air temperatures ranged from 23-37°C and 9-14°C in ambient, non-drought plots (C treatment), with an extreme maximum temperature (TMAX_EXT_ ) of 45.7°C in Nov 2014 (table S3). TMAX_AV_ and TMIN_AV_ averaged 1.4°C and 0.6°C warmer respectively in the warm (+W) treatment, with the greatest warming occurring during summer months. Drought-affected plots (+D treatment) experienced 1.9°C warming of TMAX_AV_ relative to the C treatment, with an extreme of 47.8°C, but little or no increase in daily minimums (ΔTMIN_AV_ = -0.1°C). The highest temperatures were observed in the +DW treatment (table S3), with TMAX_AV_ and TMIN_AV_ warming of 4.2°C and 0.7°C respectively. In this treatment Jan and Feb 2015 daily maximum temperatures (TMAX_AV_) exceeded 40°C, with an extreme maximum temp of 51.2°C on the hottest day. Early in the treatment period (Nov 2014), warming of TMAX_AV_ was higher in the +W than +D treatment (2.0°C vs. 1.0°C), with both independently contributing to warming in the +DW treatment (3.1°C). Over time, however, warming in +D plots increased relative to +W plots (3-5°C vs. 2-3°C).

Mid-day (11-1 pm) soil temperatures on 18 Dec 2015 (at 5 cm depth) were 25.8°C, 25.6°C, 28.2°C and 29.3°C in C, +W, +D and +DW climate treatments respectively, indicating a 2°C rise in midday temperatures associated with soil drying with a further 1°C rise associated with OTC-driven radiative forcing in +DW plots. Similar results were observed on 25 Feb (25.0°C, 25.0°C, 29.2°C and 29.4°C respectively).
